# Immunohistochemical Analysis of the Beclin-1 Expression Predicts the Progression of Oral Squamous Cell Carcinoma

**DOI:** 10.3390/ijerph182111125

**Published:** 2021-10-22

**Authors:** Ida Barca, Chiara Mignogna, Daniela Novembre, Francesco Ferragina, Maria Giulia Cristofaro

**Affiliations:** 1 Unit of Maxillofacial Surgery, Department of Experimental and Clinical Medicine, “Magna Graecia” University, Viale Europa, 88100 Catanzaro, Italy; barca.ida@gmail.com (I.B.); daniela.novembre@gmail.com (D.N.); cristofaro@unicz.it (M.G.C.); 2 Pathology Unit, Department of Health Science, Medical School, Magna Græcia University of Catanzaro, Viale Europa, 88100 Catanzaro, Italy; mignogna@unicz.it

**Keywords:** Beclin-1, maxillofacial surgery, head and neck cancer, oral cancer, oncological surgery

## Abstract

Background: Autophagy is a cellular process responsible for maintaining homeostasis; a dysregulation of this process is involved in the development and progression of neoplasms. Beclin-1 is one of the major proteins linked to autophagy. However, the data regarding the association between the role of Beclin-1 and the progression of Oral Squamous Cell Carcinoma (OSCC) are rather low. For this reason, the objective of this study is to evaluate, through immunohistochemical techniques, the prognostic role of the expression of Beclin-1 autophagy marker in patients with OSCC. Methods: This is a single-centre retrospective study that includes patients with OSCC admitted to the Maxillofacial Unit of “Magna Graecia” University between January 2019 and September 2020. All the samples obtained from surgery were treated with anti Beclin-1 antibodies and subjected to immunohistochemical methods. Results: A total of 26 samples were analysed and the following variables were evaluated for each: percentage of positive Beclin-1 expression by tumour cells, signal strength of tumour cells, and total score. The variables considered were first normalised according to the D’Agostino and Pearson test, then analysed using the Pearson linear correlation coefficient: a statistically significant correlation was found between the parameters infiltration-intensity (*p* = 0.0088), infiltration-percent (*p* = 0.0123), intensity-total score (*p* = 0.0060). Conclusions: The immunohistochemical evaluation of Beclin-1 revealed a statistically significant correlation between the intensity of the molecule’s expression and a greater degree of infiltration of the neoplasm. Beclin-1 can, therefore, be considered a valid prognostic index of disease.

## 1. Introduction

Among tumours of the oral-maxillofacial district, oral cancer is certainly the most frequent: it is the 11th most common malignancy in the world. Despite the general global trend of a slight decrease in the incidence of oral cancer, tongue cancer incidence is increasing, especially in young people [1,2]. Over 90–95% of these are Squamous cell carcinomas (OSCC), while the remainder includes adenocarcinomas, melanomas, sarcomas, and lymphomas. OSCC represents a health problem worldwide due to its morbidity and mortality. The incidence of OSCC shows variability according to the geographic location, age groups, gender, races, and ethnic groups [3,4]. Moreover, even though over the years increasingly advanced therapeutic strategies have been developed, 5-year survival rates have been reported to be about 50% [5,6], being most of them diagnosed at an advanced stage (III). As a matter of fact, in subjects affected by these neoplasms, local and regional relapses and lymph node metastases are the major causes of mortality. Therefore, early diagnosis is the most important factor affecting the overall survival and prognosis. In the last few years, numerous studies have been conducted to investigate the biological factors involved in the progression of malignancies [7,8]. Autophagy is one of the most studied. It mainly plays an adaptive role, and it is a genetically programmed cellular process responsible for maintaining homeostasis; it allows the degradation of proteins and organelles no longer needed by the cell. Scientific evidence has shown that autophagy dysregulation is involved in the development and progression of neoplasms [9,10,11,12]. Numerous studies have also highlighted how the decrease in autophagic activity is correlated with the malignant progression of numerous neoplasms [9,12,13,14,15]. One of the main proteins related to autophagy is Beclin-1 [8,16,17,18]. Beclin-1 is physiologically present in the cell and its activation initiates the autophagic process [19,20,21,22]. However, the data regarding the association between the role of Beclin-1 and the progression of OSCC are rather low. For this reason, the objective of this study was to evaluate, through immunohistochemical techniques, the prognostic role of the expression of the Beclin-1 autophagy marker in patients with squamous cell carcinoma of the oral cavity.

## 2. Materials and Methods

This single-centre retrospective study includes all patients with OSCC admitted to maxillofacial unit of University “Magna Graecia” of Catanzaro. They were all evaluated between January 2019 and September 2020.

### 2.1. Endpoint

The endpoint of this work is to evaluate, using immunohistochemical techniques, the prognostic role of the Beclin-1 expression (autophagy marker) in patients with OSCC.

### 2.2. Inclusion and Exclusion Criteria

The inclusion criteria were: (1) patients over 18years of age; (2) histological diagnosis of OSCC; (3) radical tumorectomy and neck dissection surgery; (4) absence of neoadjuvant radiotherapy treatment.

Exclusion criteria were: (1) use of adjuvant and neoadjuvant therapies; (2) no neck dissection.

All tumours were classified histologically according to the TNM classification. Biopsy samples of tumour tissue were analysed by the pathological anatomy unit of the Magna Graecia University of Catanzaro, Italy.

### 2.3. Immunohistochemical Techniques

**Figure 1:**
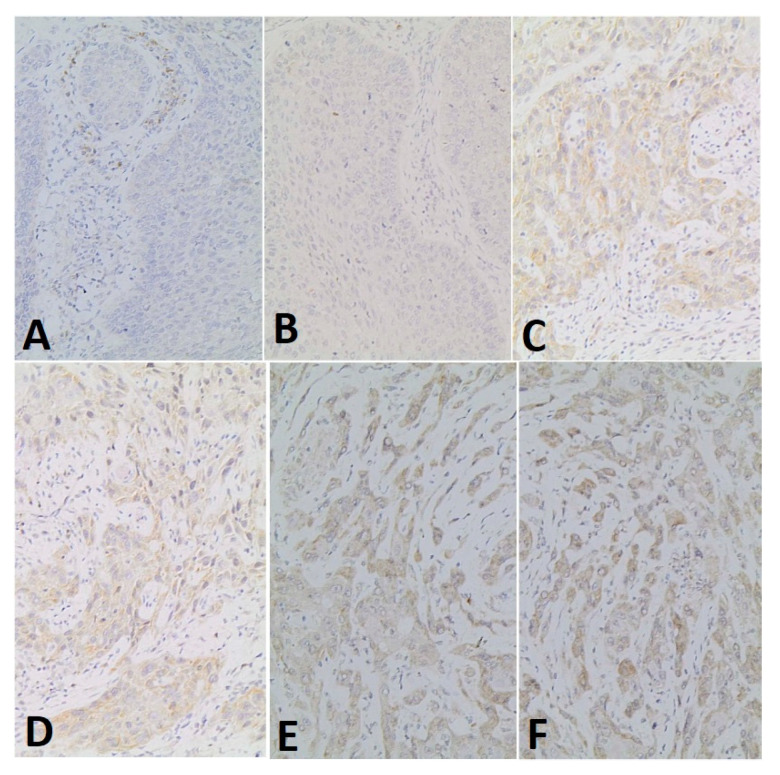
Percentage-based approach. The panel shows the score assigned based on the percentage of positive expression of Beclin-1 by tumor cells (20× magnification). (**A**) 0 as no positive cell; (**B**) 1 as <1%; (**C**) 2 as 1–10%; (**D**) 3 as 10–33%; (**E**) 4 as 33–66%; (**F**) 5 as 66–100%.

Serial sections (4μ) were obtained from the paraffin blocks for haematoxylin and eosin staining procedures and immunohistochemical techniques. For immunohistochemical investigations the deparaffinized sections were incubated with an anti Beclin-1 antibody (ab217179) (rabbit anti-human polyclonal antibody, dilution 1:150, Abcam). The standard immunohistochemical method was carried out by means of an automatic immunostainer (DAKO autostainer). The immunostainer employs a biotin-free detection system. Finally, the sections were contrasted with Mayer’s haematoxylin at 0.1%. The sections treated with these procedures were then observed under the optical microscope. Samples were examined under aZeiss Axio Imager A2 m microscope (Carl Zeiss AG, Oberkochen, Germany). The evaluating pathologist was blinded to the study groups. A semi-quantitative analysis was performed, with the evaluation of both the percentage of AURKA-positive cells and staining intensity, using score system by Allred et al. [23,24,25]. The staining results were determined for all the patients considered in the study, by checking for Beclin-1 positive cells number and the staining intensity, calculated in 10 fields from two slides (200 × magnification). A proportion score was assigned representing the estimated proportion of positively stained tumour cells (0 as none, 1 as <1%, 2 as 1–10%, 3 as 10–33%, 4 as 33–66%, 5 as 66–100%) (Figure 1).

**Figure 2:**
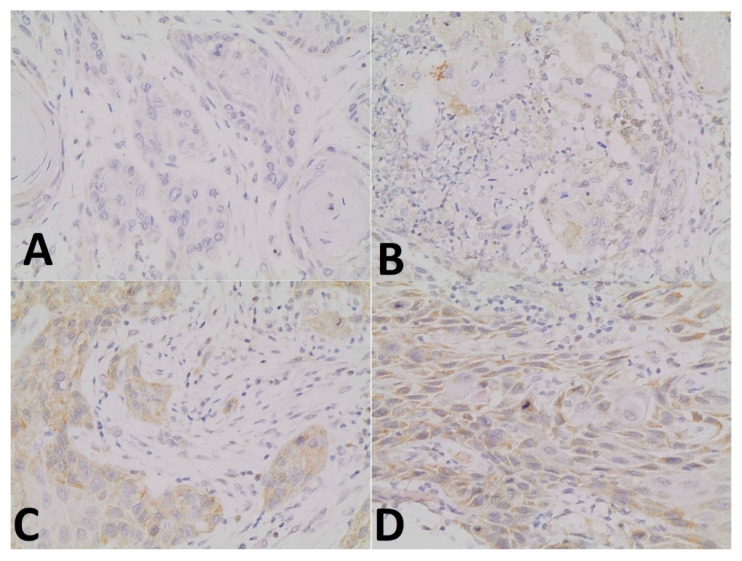
(20× magnification): Intensity-based approach. The panel shows the score assigned to the four different signal intensities: (**A**) 0 as none; (**B**) 1 as weak; (**C**) 2 as average intensity; (**D**) 3 as strong intensity.

Average estimated intensity of staining in positive cells was assigned as an intensity score (0 as none; 1 as weak; 2 as intermediate; 3 as strong) (Figure 2).

An immunoreactive score ranging from 0 to 8 was defined as the sum of proportion score and intensity score.

The colour reaction was observed under a microscope, and photographs of the slides were then acquired. Five high-power visual fields were randomly selected with 100 cells in each field. Samples were examined under aZeiss Axio Imager A2 m microscope (Carl Zeiss AG, Oberkochen, Germany). The evaluating pathologist was blinded to the study groups.

### 2.4. Statistical Analysis

Statistical analysis was performed using the GraphPad program (GraphPad Company, San Diego, CA, USA). Spearman’s linear correlation coefficient rho (⍴) was used, evaluating the existence of a correlation between the parameters: neoplastic infiltration and Beclin-1 signal intensity, neoplastic infiltration and percentage of positive cells, infiltration, and total score (sum of intensity and percentage). The *p*-value was then obtained: the accepted significance level was set at *p* < 0.05.

## 3. Results

Fifty-three patients underwent surgery under general anaesthesia for OSCC. Of these, only 26 (49.06%) were included in the inclusion criteria: 15 women (57.69%) and 11 men (42.31%) with an average age of 67.61 years-old (range 26 to 90 years). A total of 25 patients (96.15%) had primary tumours, only 1 patient (3.85%) was suffering from recurrences; no one had secondary tumour. None of these underwent neoadjuvant chemotherapy and/or radiotherapy. In a total of 11 patients (42.31%) the disease was diagnosed at stage 1, in 8 patients (30.77%) at stage 2, in 3 patients (11.54%) at stage 3, in 4 patients (15.38%) at stage 4. Precisely, 11 staged as T1N0M0 (42.31%), 1 staged as T1N2bM0 (3.85%), 8 staged as T2N0M0 (30.77%), 2 staged as T2N1M0 (7.68%), 3 staged as T2N2bM0 (11.54%), 1 staged as T3N0M0 (3.85%). No patient had distant metastases. All patients were treated with radical tumorectomy; various reconstructive techniques were used: 4 local flaps (15.38%; buccal fat pad flap, buccinator myo-mucosal flap) and 22 primary closures (84.62%). Neck dissection was performed in 23 cases (88.46%); in the remaining 3 cases (11.54%), the patient refused this method given the very advanced age. Twenty patients needed Intensive Care Unit (ICU) after surgery for controlled awakening. All collected samples were treated with Beclin-1 and the mean infiltration was 8.02 mm. The following variables were evaluated: percentage of positive expression of Beclin-1 by tumour cells, signal intensity of tumour cells, total score. Regarding the signal strength: a single sample had no intensity (0; 3.85%), thirteen samples had weak intensity (1; 50%), nine samples had average intensity (2; 34.61%) and three samples had strong intensity (3; 11.54%). Regarding the percentage of positive expression of Beclin-1 by tumour cells: a single sample had no positive cells (0; 3.85%), seven samples had a percentage <1% (1; 26.92%), four samples had a percentage between 1% and 10% (2; 15.38%), eight samples had a percentage between 10–33% (3; 30.77%), four samples ha a percentage between 33% and 66% (4; 15.38%), two samples had a percentage between 66% and 100% (5; 7.7%). Data of the semiquantitative analysis of processed tumour samples are summarised in Table 1.

Considered variables were normalized according to the D’agostino and Pearson test. Therefore, Pearson’s linear correlation coefficient was used: a statistically significant correlation was found between the parameters infiltration-intensity (*p* = 0.0088), infiltration-percent (*p* = 0.0123), and intensity-total score (*p* = 0.0060). 

## 4. Discussion

The oral-maxillofacial area is composed of multiple tissues from which a huge number of cancers can originate. OSCC is surely one of the most common cancers in the world. Although remarkable progress has been made in treatment modalities over the years, the five-year survival rate is lower than that of other solid cancers [26]. This could be due both to the lack of early diagnosis markers and to the resistance of many patients to chemotherapy drugs [27]. Therefore, the determination of those molecules involved in cell survival pathways is very important for making an early diagnosis. Autophagy is a highly regulated process of degradation and recycling of cellular constituents, which is fundamental in maintaining homeostasis. Beclin-1 is a cellular protein involved in this process, the activation of which aims to safeguard the survival of the cell. From the data collected in the literature, it is clear that Beclin-1 is related to the onset of neoplasms in different districts [28,29,30,31,32,33,34]. More and more evidence shows the importance of autophagy in regulating of cancers development and progression and in determining the response to anticancer therapy [35]. However, the data regarding the association between the role of Beclin-1 and the progression of OSCC are rather scarce. For this reason, the objective of this study is to evaluate, through immunohistochemical techniques, the prognostic role of the expression of the Beclin-1 autophagy marker in patients with OSCC. According to Wang et al. the increase in autophagy, expressed as an increase in Beclin-1, is a tumour suppression factor in squamous cell carcinomas of the tongue. In particular, the reduced presence of Beclin-1 is associated with the progression of the disease and, therefore, with a worse prognosis [36]. More recently, Kapoor et al. [35] observed the decrease in mRNA levels in tumour tissue compared with healthy peritumour tissue. However, the increase in autophagy can promote the progression of tumours, in fact many studies show that this process provides sufficient nutrients that allow the growth of cancer cells. In fact, some authors have found that in breast cancer the increase in autophagy induces the interaction between Beclin-1 and HER2 with consequent tumorigenesis [37]. This is the reason why we decided to examine, with a semi-quantitative method widely validated in the literature [38,39,40], the immunohistochemical expression of the Beclin-1 product. Our results, unlike the data in the literature, show that the increase in Beclin-1 expression is correlated with the degree of tumour infiltration (expressed in mm). This points out that Beclin-1 acts as a tumorigenesis promoter in the OSCC. Furthermore, autophagy activated by Beclin-1 plays a crucial role in anticancer therapy: the resistance of cancer cells to some chemotherapy drugs enhances the autophagic process itself, increasing the survival of cancer cells. Accordingly, it is reasonable to take on that increased Beclin-1 expression in OSCCs may be associated with disease progression; it can also contribute to greater tumour infiltration through the still unclear role of autophagy during the development of cancer. Autophagy, therefore, has a promoting role in the evolution of the OSCC. In literature, it is reported that the presence of Beclin-1 in nuclei is related to the ability to repair DNA damage induced by radiation. In our study, we demonstrate a statistically significant correlation between the immunohistochemical signal of Beclin-1 (within the nucleus) and the infiltration of the neoplasm.

Therefore, further studies would be necessary, which led us to continue to understand the molecular alterations of the autophagic process involved in the progression of OSCC and its potential as a therapeutic target.

## 5. Conclusions

Squamous cell carcinoma of the oral cavity is a rather frequent cancer, with variable prognosis based on the location, grading, and spread of the disease. In conclusion, the immunohistochemical evaluation of Beclin-1 revealed a statistically significant correlation between the intensity of the molecule’s expression and a greater degree of infiltration of the neoplasm. All this, in turn, is associated with a greater probability of developing distant metastases and consequently of having a worse prognosis of the disease. Therefore, an indispensable condition for therapeutic success (in terms of survival of patients with OSCC) is a diagnosis as early as possible, as well as the evaluation of a valid prognostic index that can direct towards the most appropriate therapeutic treatment. Beclin-1 can, therefore, be considered a valid prognostic index of disease. However, given the small number of the sample, this remains a preliminary study; future investigations are underway.

## Figures and Tables

**Table 1:** Sample characteristics.

*N*°	Sex	Age	TNM	Neoplastic Infiltration	Beclin-1 Signal Intensity	Percentage of Positive Cells	Total Score
*1*	F	50	T2 N0 M0	10 mm	1	3	4
*2*	F	76	T1 N2b M0	18 mm	1	1	2
*3*	F	71	T2 N1 M0	2 mm	1	1	2
*4*	M	70	T1 N0 M0	7 mm	1	1	2
*5*	M	26	T1 N0 M0	7 mm	2	2	4
*6*	F	75	T1 N0 M0	8 mm	2	4	6
*7*	M	72	T1 N0 M0	2.5 mm	3	5	8
*8*	M	82	T1 N0 M0	1 mm	3	5	8
*9*	F	85	T1 N0 M0	4 mm	1	1	2
*10*	F	60	T2 N0 M0	5 mm	2	4	6
*11*	M	54	T2 N1 M0	10 mm	1	2	3
*12*	F	80	T1 N0 M0	11 mm	1	1	2
*13*	F	75	T3 N0 M0	10 mm	2	4	6
*14*	F	77	T2 N0 M0	5 mm	1	3	4
*15*	F	75	T2 N0 M0	4 mm	2	3	5
*16*	M	45	T1 N0 M0	11 mm	2	3	5
*17*	M	46	T2 N2b M0	12 mm	2	3	5
*18*	F	35	T1 N0 M0	11 mm	2	3	5
*19*	M	69	T2 N2b M0	18 mm	0	0	0
*20*	M	87	T2 N0 M0	7 mm	1	2	3
*21*	M	59	T2 N0 M0	18 mm	1	1	2
*22*	F	50	T1 N0 M0	9 mm	1	1	2
*23*	F	74	T2 N2b M0	5 mm	1	4	5
*24*	M	92	T2 N0 M0	3 mm	3	3	6
*25*	F	93	T1 N0 M0	7 mm	1	3	4
*26*	F	80	T2 N0 M0	3 mm	2	2	4

## Data Availability

The data presented in this study are available on request from the corresponding author.
